# Vitreous Antioxidants, Degeneration, and Vitreo-Retinopathy: Exploring the Links

**DOI:** 10.3390/antiox9010007

**Published:** 2019-12-20

**Authors:** Emmanuel Ankamah, J. Sebag, Eugene Ng, John M. Nolan

**Affiliations:** 1 Nutrition Research Centre Ireland, School of Health Science, Carriganore House, Waterford Institute of Technology, West Campus, Co., X91 K236 Waterford, Ireland; eugene@ioes.ie; 2 Institute of Eye Surgery, UPMC Whitfield, Buttlerstown, Co., X91 DH9W Waterford, Ireland; 3 VMR Consulting Inc., Huntington Beach, CA 92647, USA; JSebag@vmrinstitute.com

**Keywords:** antioxidants, vitreous, oxidative stress, vitreous degeneration, floaters, vision degrading myodesopsia

## Abstract

The transparent vitreous body, which occupies about 80% of the eye’s volume, is laden with numerous enzymatic and non-enzymatic antioxidants that could protect the eye from oxidative stress and disease. Aging is associated with degeneration of vitreous structure as well as a reduction in its antioxidant capacity. A growing body of evidence suggests these age-related changes may be the precursor of numerous oxidative stress-induced vitreo-retinopathies, including vision degrading myodesopsia, the clinically significant entoptic phenomena that can result from advanced vitreous degeneration. Adequate intravitreal antioxidant levels may be protective against vitreous degeneration, possibly preventing and even improving vision degrading myodesopsia as well as mitigating various other vitreo-retinopathies. The present article is, therefore, a review of the different antioxidant molecules within vitreous and the inter-relationships between vitreous antioxidant capacity and degeneration.

## 1. Introduction

### Ocular Antioxidants—Protection against Oxidative Damage and Disease

Vision relies on the coordinated roles played by various structures of the visual system, from the tear film on the ocular surface to the visual centers within the brain. Visual perception commences with sensory information organization, the process by which the highly specialized neurosensory retina of the eye captures photons from the environment and converts them into neural signals for visual processing and transmission to the higher visual centers within the brain [1]. Concurrently, the eye is exposed to exogenous, potentially injury-precipitating factors including visible light, ultraviolet light, ionizing radiation, and environmental toxins; as well as endogenous stress-inducing influences generated by the mitochondria within ocular tissues during the eye’s physiological functions [2]. These endogenous and exogenous oxidants produce unstable reactive oxygen species (ROS) characterized by one or two unpaired electrons within their external orbit [3]. 

While normal concentrations of ROS are a physiological response to stress and are an integral part of normal ocular metabolic activity, excess levels could be debilitating to the eye [4]. To remain functional, the eye is replete with an assortment of antioxidants (substances that, when present in low concentrations compared to that of an oxidizable substrate, significantly delay or inhibit the oxidation of the substrate) by which it mitigates the damaging effects of ROS [5]. Over-production or inadequate elimination of ROS beyond the counteracting ability of the eye’s antioxidant system can cause ocular tissues to be overwhelmed, a phenomenon referred to as oxidative stress [6]. The pathological cascade following oxidative stress are ocular physiologic dysfunction, ocular tissue death and consequently, ocular degenerative disorders [7]. 

Emerging evidence suggests a relationship between decreased intraocular antioxidant capacity and the onset of ocular diseases such as endothelial Fuch’s dystrophy, cataract, age-related macular degeneration (AMD), and diabetic retinopathy (DR) [2,8,9]. Also, aging, toxins, inflammations, infections, and possibly nutritional imbalance deplete intraocular antioxidants, necessitating a constant supply of antioxidants via diet or supplementation [10]. It is thus not surprising to observe recent strides in research focused on the application of antioxidants as plausible therapeutic and prophylactic agents in the management of ocular diseases, an idea which forms a critical aspect of personalized medicine and an important future direction in healthcare [11,12]. 

Conventional medicine, as has been practiced over the years, is a disease-oriented and reactive approach of treating patients’ complaints as well as ensuring that clinically measured disease-related indices are normalized. This approach of “disease care” appropriates the majority of health resources to the management of clinical manifestation of severe pathologies, and fails to address the entirety of health, which is a state of complete physical, mental and social well-being, and not just the absence of disease [13,14]. 

Personalized medicine, a predictive, preventive, and individual-specific approach to healthcare, on the other hand, focusses on identifying distinct profiles of a person’s health: genetic, biological, and environmental, with the ultimate goal of either avoiding the manifestation of diseases in individuals or providing treatments customized to the person in question [15]. Facilitated by the constant innovations in biochemical, genomic, and diagnostic apparatus, the trajectory towards personalized medicine will involve everything ranging from lifestyle modifications (physical exercise and dietary or nutritional prescriptions), health promotion campaigns, screening exercises, predictive algorithms to isolate individuals with high risk of disease, telemedicine monitoring and assessment, early and appropriate diagnosis, to genetic-tailored therapies for diseases. Not only will this preventive and specific healthcare delivery improve patient well-being but also reduce the financial burden on patients and healthcare systems [16]. Thus, dietary supplementation with antioxidants, aimed at mitigating oxidative stress and injury, may subserve the preventive aspects of personalized medicine, including eye care. 

Antioxidant molecules within some ocular structures namely aqueous humour, cornea, crystalline lens, and retina have been reviewed elsewhere [2,17]. However, there is little information on the antioxidant profile of the vitreous body [2,18]. The present review therefore examines the various antioxidant molecules within vitreous and how their depletion might influence vitreous degeneration and the pathogenesis of vitreo-retinal diseases. Evidence from reviews, metabolomic, and proteomic studies of vitreous was examined, and the identified vitreous antioxidant molecules will be discussed.

## 2. The Vitreous Body

### A Look at the Vitreous Body and Not just through It

Historically, vitreous was noted mainly for its contribution to intraocular clarity and to the maintenance of the globe’s shape. As a result, no physiological importance was ascribed to vitreous, further fueled by decades of experience in surgically removing vitreous without apparent detriment to ocular health [19,20]. However, enabled by advancements in vitreous imaging and biochemical analyses, recent studies have provided invaluable insights into the molecular constitution of this seemingly invisible organ, and its contribution to ocular health and disease [21,22]. Thus, it has become imperative for eye care professionals to critically observe vitreous while examining their patients and for scientists to consolidate efforts at enhancing our understanding of vitreous in healthy and diseased states. The current disposition is succinctly captured in a quote by Prof. J. Sebag who admonishes to “*look at vitreous and not just through it* [21].” 

**Figure 1:**
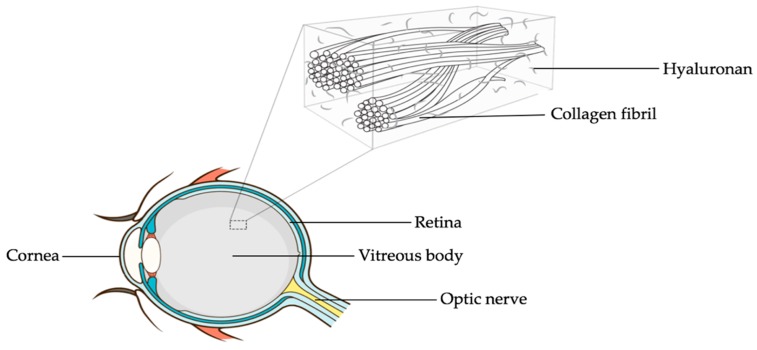
Cross-sectional diagram of the human eye showing the vitreous body and the interaction between its two principal components, collagen and hyaluronan. Courtesy of Emmanuel Ankamah.

A look at the vitreous body reveals that it is the largest ocular structure filling the space within the posterior segment bordered by the posterior lens surface and the inner limiting membrane (ILM) of the retina. With a total volume of approximately 4 mL, the vitreous body is mainly composed of water (about 98%–99%), collagen fibres, glycosaminoglycans (GAGs; predominantly hyaluronan), non-collagenous proteins (including opticin and versican), and small amounts of trace metals and elements [23,24]. The gel nature of vitreous is attributed to the interaction between its two principal components, collagen and hyaluronan (Figure 1). 

The vitreous body is subdivided into 3 broad anatomical regions: vitreous cortex (anterior and posterior), central vitreous, and vitreous base. The vitreous cortex is a lamellar structure attached to the ILM of the retina posterior to the peripheral vitreous base by an extracellular matrix “adhesive” consisting of fibronectin, opticin, laminin, heparan sulfate, and chondroitin sulphate. Anteriorly, the vitreous cortex is attached to the lens [25]. Vitreous is relatively acellular with only a monolayer of mononuclear phagocytes, hyalocytes, located within the posterior vitreous cortex, about 50 µm from the ILM [26]. Vitreous contributes to intraocular media clarity, the regulation of intraocular oxygen tension, and the maintenance of intraocular pressure [27]. It also confers protection by acting as a shock absorber, done by the collagen fibres which reduce the compressive forces of hyaluronan (HA) when the globe is exposed to external pressure [28,29]. Vitreous acts as a reservoir for nutrients and metabolites that it receives from synthesis within the non-pigmented ciliary epithelium and retinal pigment epithelium [19,30,31,32]. Hyalocytes play a vital role in modulating intraocular inflammation in non-inflamed eyes, thereby contributing to intraocular transparency [26]. 

Collagen concentration within the human vitreous body approximates to 300 µg/mL, accounting for 0.5% of the total vitreous protein [33]. Vitreous assembles collagen fibres in a heterotypic fashion, organizing collagen types II, V/XI, VI, and IX, with collagen II being the most abundant. Vitreous collagen fibrils are thin and unbranched with uniform diameter ranging between 10 to 20 nm (depending on the species) [34]. Collagen constitutes the essential structural component of vitreous and its removal in vitro results in vitreous liquefaction [19,35]. Hyaluronan, a polydisperse polysaccharide, is the predominant GAG within vitreous [36]. The concentration of HA within vitreous ranges between 0.02–1 mg/cm^3^ [37]. As the primary mediator of the internal adhesivity of vitreous, HA plays a synergistic role with collagen and other proteoglycans in regulating the stiffness of vitreous [38]. 

As a connective tissue matrix, vitreous shares similar biochemical properties with the synovial tissue around joint spaces. Both vitreous and synovial fluid are viscoelastic tissues consisting mainly of collagen and HA. Vitreous collagen type II, however, differs slightly in chemical composition due to the presence of terminal peptide constituents in its collagen [39]. Particular to vitreous and cartilage is an acidic glycoprotein with a five-armed configuration, *cartilage oligomeric matrix protein* [40]. Its function in vitreous is, however, yet to be identified. Vitreous and synovial fluid separate tissues and protect against friction and high-frequency stresses [41]. The similarities in macromolecular structure between vitreous and joints is the underlying explanation as to why both tissues show characteristic clinical manifestations in inherited collagen disorders such as Marfan and Ehlers–Danlos syndromes. 

## 3. Vitreous Degeneration

Two principal and inter-related processes account for age-related degeneration of the vitreous body: liquefaction (synchysis senilis) and vitreo-retinal dehiscence, which in combination result in posterior vitreous detachment [42]. Liquefaction is a physico-chemical degenerative change that disrupts the homogeneity of the gel vitreous. It is characterized by dissociation of HA from collagen, aggregation of collagen fibrils, and formation of lacunae (collagen-free spaces filled with liquid) within the vitreous body [43,44]. There is also evidence suggesting collagen degradation as a mechanism for vitreous gel liquefaction [45]. Concurrent with gel liquefaction, vitreo–retinal adhesion at the posterior and equatorial vitreous begins to weaken. Further degeneration results in separation of the posterior vitreous cortex (PVC) from the ILM of the retina, a phenomenon referred to as posterior vitreous detachment (PVD) [46,47]. 

Vitreous degeneration commences quite early in life, with 12.5% of the vitreous gel being liquified by age 18 years. After increasing during growth and development, the volume of the gel remains stable until about the fifth decade when it begins to decrease in parallel with an increase in liquid vitreous [48,49]. Significant vitreous degeneration translates into an entoptic phenomenon, vitreous floaters, which is the perception of hair-like, fly-like, gray, linear images like cobwebs, primarily within the central visual field [9]. When vitreous floaters cause subjective disturbance in vision and objective impairment of contrast sensitivity function, the diagnosis of vision degrading myodesopsia is applicable [50]. Vitreous degeneration has been shown to correlate with increased intravitreal oxidative stress biomarkers and proteolytic enzymes [51,52]. The total reactive antioxidant potential of human vitreous decreases with age and with oxidative-stress-induced retinal pathology [53,54,55]. Lastly, vitreous degeneration with associated loss of antioxidative potency increases exposure of the natural crystalline lens to oxygen and ROS, promoting the progression of nuclear cataracts [27].

### 3.1. Vitreous Liquefaction

While the causative mechanisms for vitreous liquefaction have not been fully unraveled, our current understanding of this process can be summed up in two categories: oxidative-stress-induced liquefaction and enzyme-induced liquefaction [52,56]. 

#### 3.1.1. Oxidative-Stress-Induced Liquefaction 

ROS have been proposed to be the main cause of vitreous structure alteration in aging [57]. Liquefaction has been reported with in vivo and in vitro animal model experiments investigating the effect of free radicals, generated from photosensitizer and white-light irradiation, on vitreous [57,58,59,60]. Light-induced free radicals have also been shown to decrease the molecular weight of HA, induce HA depolymerization and, consequently, liquefaction [18,57,61,62]. Liquefaction caused by light-induced ROS has been described to be age-related [59]. This could be because riboflavin, the naturally present photosensitizer molecule within vitreous, is irradiated by white light on a daily basis during the course of a lifetime. This results in an age-dependent build-up of free radicals that contribute to the molecular alteration of vitreous collagen and HA [57]. 

#### 3.1.2. Enzymatic Liquefaction

A number of proteolytic enzymes have been implicated in vitreous gel liquefaction. The mechanisms of action of these enzymes can be understood by observing their effects on collagen and HA. Although contrasting views abound in the literature regarding the fate of collagen and HA with enzymatic activity, the evidence points to proteolytic enzymatic activity as a mechanism for vitreous liquefaction.

i. Enzyme Effects on Vitreous Collagen: There is evidence to suggest that increased enzyme activity causes liquefaction either by collagen cleavage or collagen degradation. Vaughan and associates have reported an increase in the level of the enzyme plasmin(ogen) in vitreous with age [52]. Plasminogen activates a matrix metalloproteinase (MMP), progelatinase or proMMP-2, which results in the cleavage of collagen and subsequently, vitreous liquefaction [52]. While it is true that the observation of an increase with age is only an association and not proof of causation, this is an avenue of research to pursue. Góes et al. reported degradation of the chondroitin sulphate chains of collagen IX after rabbit vitreous was treated with chondroitin ABC lyase [63]. A morphological change of collagen IX diminishes the shielding effect it renders to the surface of collagen II. This makes collagen II fibres susceptible to lateral fusion, fibrillar aggregation, and liquefaction [64]. It is not clear, however, that chondroitin ABC lyase is active in the human vitreous body. Bioanalytical studies found that collagenase and trypsin degrade collagen type II in vitro, altering the mechanical behavior of vitreous and inducing liquefaction [65,66,67]. This has been confirmed by experimental models of the aging eye, which have shown increased liquefaction, reduced vitreous viscosity, and decreased elasticity, after intravitreal treatment with the active enzyme collagenase [65,66]. Also, Los and colleagues observed collagen fragmentation in the lacunae of vitreous undergoing liquefaction and attributed the cause of this phenomenon to active enzyme activity within the aging vitreous [45]. 

ii. Enzyme Effects on Vitreous Hyaluronan: The reported effect of enzyme activity on HA are equivocal. A study by Bishop and colleagues reported only a reduction in vitreous gel wet weight, but not a destruction of its gel state, following complete depolymerization of HA by streptomyces HA lyase, chondroitin ABC lyase, and testicular hyaluronidase. Other studies, on the contrary, have reported vitreous liquefaction following extensive HA depolymerization by hyaluronidase [59,68,69].

### 3.2. Posterior Vitreous Detachment 

Significant vitreous liquefaction (synchysis) with simultaneous vitreo-retinal interface dehiscence, results in innocuous PVD, characterized by the complete separation of the vitreous cortex from the ILM of the retina in all areas posterior to the vitreous base [42]. Evidence from autopsy studies has indicated a 51% prevalence of PVD in the seventh decade, which increases further to 63% in the eighth decade [43]. Aging aside, high myopia, menopause, and hereditary extracellular matrix syndromes such as Stickler syndrome, Ehlers–Danlos syndrome, and *LAMA5* multisystem syndrome, are other known risk factors for PVD [70,71,72,73]. PVD occurs earlier in higher myopes compared to emmetropes, owing in part to precocious liquefaction of myopic vitreous [72,73]. The higher incidence of PVD in postmenopausal women has been attributed to hormonal changes associated with menopause (i.e., lowered levels of estrogen in postmenopausal compared to premenopausal women) [71]. Although innocuous PVD is usually not sight-threatening, there are often visual complaints associated with this phenomenon, notably floaters and photopsia or Moore’s light flashes, which are the perception of sudden flashes of light [42]. 

In the case where firm vitreo-retinal adhesions persist in the face of significant vitreous synchysis, anomalous PVD (APVD) can occur [74,75]. This unifying concept in vitreo-retinopathies is based upon the premise that same initiating abnormality (i.e., excess liquefaction without concurrent vitreo-retinal dehiscence) can explain several seemingly disparate vitreo-retinal disorders ranging from retinal tears/detachments to axial vitreo-macular traction syndrome and tangential vitreo-maculopathies such as macular pucker and macular holes. APVD has also been observed in eyes with proliferative diabetic retinopathy, Eale’s disease, high myopia, and congenital collagen disorders such as Ehlers–Danlos syndrome [76,77,78,79]. APVD can be classified as full-thickness, when the entire PVC remains attached to the retina at specific locations, or partial-thickness where the PVC splits (referred to as “vitreoschisis”), with its outer layer adhering to the retina [80]. Clinical manifestations include rhegmatogenous events (when traction is exerted on the peripheral retina), vitreo-papillopathies (when traction is exerted at the optic disc), neovascularization and vitreous haemorrhage in ischemic retinopathies (when traction is on abnormal blood vessels arising from the retina and/or optic disc), and the two aforementioned forms of vitreo-macular traction [47,80,81]. At the level of the macula, vitreoschisis results in macular pucker, when the split occurs anterior to the level of the hyalocytes and vitreous is separated from the optic disc, or macular hole, when the split occurs posterior to the hyalocytes and vitreous remains attached to the optic disc [82]. There are also cases of macular hole that do not involve vitreoschisis, although better imaging technologies are needed to fully characterize these vitreo-maculopathies. 

The working hypothesis that derives from the foregoing is that vitreous degeneration is an alteration of vitreous extracellular matrix biology associated with an age-related decrease in vitreous antioxidant capacity and concurrent increase in intravitreal ROS and proteolytic enzymes, and is a potential precursor for vitreo-retinal pathologies such as vitreo-maculopathies and retinal tears/detachments [73,74,79]. Interestingly, such vitreo-retinal pathologies can elevate serum and tissue levels of biochemical stress factors like neuron specific enolase (NSE) found in the eyes and serum of patients with retinal detachment [83]. This is not surprising, as retina is an extension of the central nervous system (CNS). As a further corollary to the CNS, reattachment of the neurosensory retina following retinal detachment repair can trigger hyperoxia and increased free radical generation, culminating in reperfusion injury similar to CNS strokes [84]. Thus, following surgical restoration of vitreo-retinal structure, new strategies need to be forged to improve visual function. Vitreous antioxidant activity could mitigate reperfusion injury effects, unless depleted due to vitreous degeneration and/or disease. Vitreous degeneration and loss of antioxidant activity can also exacerbate diabetic retinopathy and age-related macular degeneration [76,85,86,87,88,89,90,91,92]. 

Antioxidants have been shown to scavenge ROS and inhibit the activities of proteolytic enzymes [62,93]. Thus, vitreous antioxidants may be protective against vitreous degeneration and oxidative-stress-induced retinal pathology. Given the apparent importance, there is a need to profile vitreous antioxidants in a bid to understand their role in vitreous health, degeneration, and vitreo-retinal diseases.

## 4. Vitreous Antioxidant Profile in Health and Disease

**Figure 2:**
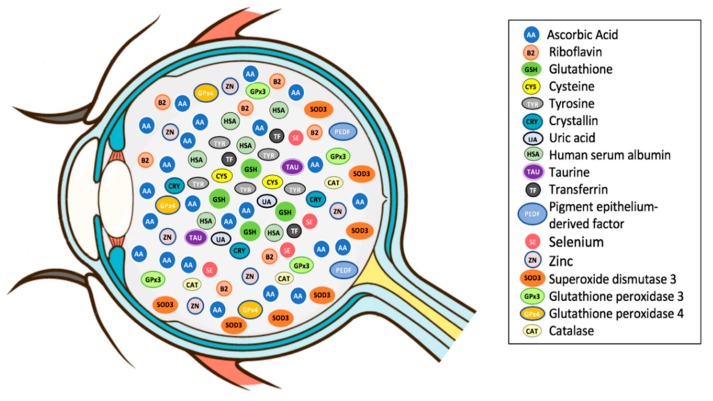
Diagram showing the antioxidants within the human vitreous. Courtesy of Emmanuel Ankamah.

**Figure 3:**
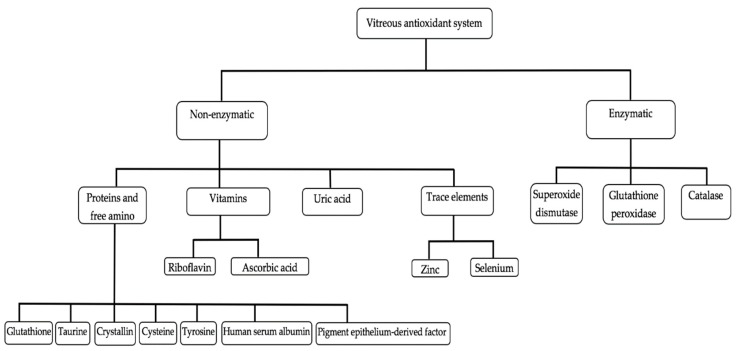
Classification of vitreous antioxidants. Courtesy of Emmanuel Ankamah.

As previously defined, an antioxidant is a substance that, when present in low concentration compared to that of an oxidizable substrate, significantly delays or inhibits the oxidation of the substrate [5]. Vitreous accumulates a high concentration of hydrosoluble antioxidants, which could protect the eye from oxidative stress (Figure 2; Table 1) [94]. Vitreous antioxidants can be broadly classified into enzymatic and non-enzymatic antioxidants (Figure 3).

### 4.1. Non-Enzymatic Vitreous Antioxidants

Antioxidants within this class comprise non-enzymatic molecules that are capable of rapidly inactivating radicals and oxidants [95]. Based on the source of non-enzymatic vitreous antioxidants, they can be classified into metabolic and nutrient non-enzymatic antioxidants. Metabolic antioxidants are endogenous antioxidants produced by the body and include glutathione, metal-chelating proteins, uric acid, and transferrin. Nutrient antioxidants include the class of non-enzymatic antioxidants that are exogenously sourced through foods and supplements, for example, vitamin C, vitamin B2, and trace metals (zinc and selenium) [96]. 

#### 4.1.1. Vitamins

i. Vitamin C: Also referred to as ascorbic acid (AA), Vitamin C is a water-soluble molecule present in most tissues in its anionic state [17]. Humans cannot synthesize AA de novo and source this molecule exogenously [97]. The vitreous gel receives its supply of AA from plasma by active transport from the ciliary process of the ciliary body [98]. AA concentration within the vitreous body approximates to 2 mmol/L, about 33 times higher than plasma concentration [99]. Also, AA within intact gel vitreous is higher than in liquefied vitreous and in vitreous of proliferative diabetic retinopathy (PDR) patients [27,100]. As an antioxidant, AA is oxidized in order to convert superoxide anions and lipid hydroperoxidases into stable forms, thereby preventing lipid peroxidation, the oxidative damage of lipids. AA consumes oxygen released at the vitreo–retinal interface, in an ascorbate-dependent fashion, and guards against intraocular oxidative stress and nuclear cataract development [27]. AA also functions as an intrinsic modulator of hyalocyte proliferation and of extracellular matrix production by hyalocytes [101,102]. AA serves as an enzyme co-factor to a number of enzymes, especially hydroxylases, which are involved in collagen synthesis [103].

ii. Vitamin B_2_: Riboflavin has been detected in both human and animal vitreous, with 0.8 µg/100 mL and 8.0 µg/L average concentrations detected in the ox and bovine vitreous, respectively [104,105]. Riboflavin plays an essential role in the glutathione redox cycle and guards against lipid peroxidation [106]. Riboflavin acts as the precursor for two coenzymes, flavin mononucleotide (FMN) and flavin adenine dinucleotide (FAD), which are involved in energy metabolism. FAD is essential for the activity of glutathione reductase, which converts oxidized glutathione (GSSH) into reduced glutathione (GSH) (see discussion of GSH below) [107]. Also, riboflavin functions as an antioxidant through the oxidation of dihydroriboflavin, the reduced form of riboflavin, to produce reducing equivalents for the deactivation of hydroperoxides [108]. Dihydroriboflavin also protects against reperfusion oxidative damage by reducing oxidized iron in hemeproteins [109,110]. Riboflavin directly scavenges for free radicals produced by mutagens, thereby inhibiting their mutagenicity [111]. For a review of the antioxidant ability of riboflavin, see [106,107]. On the other hand, riboflavin is a photosensitizer which can mediate a riboflavin-sensitized photochemical reaction and result in age-related liquefaction of vitreous [57]. Thus, therapeutic use of riboflavin for eye diseases may be a two-edged sword that needs to be wielded carefully to achieve a salubrious outcome.

#### 4.1.2. Proteins and Free Amino Acids

Proteomic analysis of human vitreous has revealed proteins and several amino acid constituents that play important roles in ocular development as well as function as antioxidants [112,113]. The majority of vitreous antioxidant proteins are located within the central vitreous, among them are glutathione, taurine, crystallin, cysteine, uric acid, tyrosine, human serum albumin, transferrin, and pigment epithelium-derived factor [114]. 

i. Glutathione (GSH): Glutathione is a cysteine-containing peptide and a thiol antioxidant with an average concentration of 0.26 mmol/L [115,116,117]. The concentration of glutathione within vitreous is relatively low compared to AA [116]. As an antioxidant, glutathione can directly remove selected oxygen radicals and indirectly assist in the recycling of vitamins C and E [118]. Also, GSH inhibits the degradation of HA by acting as a scavenger for hydroxyl radicals [119,120]. GSH is a cofactor for glutathione peroxidase activity of reducing lipid hydroperoxides, producing alcohol and GSSH in the process [107]. Reduced intravitreal GSH level has been linked with the pathological complications of inflammation and neovascularization in proliferative diabetic retinopathy (PDR) and Eales’ disease [117,121]. Other reports, on the contrary, indicate an increase in intravitreal GSH in PDR eyes [122]. This increase may represent a protective response to detoxify the diabetes-associated redox alteration of vitreous. Indeed, profound structural abnormalities have been identified in human vitreous that are independent of the effects of diabetes on the retina [76,79,123].

ii. Taurine: Taurine is a free amino acid that abounds in tissues during development [124]. Taurine has been detected in rat vitreous at a concentration of 1.72 µmol/mL [125]. Although the exact role of taurine within vitreous is yet to be elucidated, taurine, as an organic osmolyte, has been proposed to be involved in the vitreous-mediated ionic exchanges that occur between the retina and the anterior segment [126]. In addition, it has been proposed that the retina possibly receives its supply of taurine from vitreous [127,128]. Taurine provides antioxidative and neuroprotective functions to ocular tissues, although this mechanism has not been fully understood in the human eye [129]. Depletion or deficiency of taurine leads to loss of photoreceptors and can impede visual function in man and in animal models [127,130]. 

iii. Crystallin: Crystallin is a chaperone or stress protein which accumulates within the lens more than all other ocular tissues [131]. Both α- and β-crystallins have been isolated in rat vitreous [132]. β-crystallin B2 (molecular weight ~23 kDa) has been recently identified by matrix-assisted laser desorption ionization time of flight (MALDI-TOF) in normal human vitreous [133]. β-crystallin S, β-crystallin A4, β-crystallin A3, α-crystallin B chain, and γ-crystallin C have also been found in the vitreous body of both PDR patients and controls [134]. Crystallin levels were significantly lower in vitreous from PDR patients compared with controls. Crystallin performs an anti-apoptotic role by inhibiting the formation of ROS, thereby reducing oxidative stress [135]. 

iv. Cysteine: Cysteine, a non-essential amino acid with a highly reactive thiol group, is found in most peptides and proteins. Cysteine acts as the rate limiting precursor for the synthesis of GSH [18]. As an antioxidant, its reactive thiol group is oxidized to cystine disulphide and aids in maintaining a redox equilibrium within a cell, tissue, or biofluid [136].

v. Tyrosine: L-tyrosine is a monophenolic amino acid and a byproduct of the pentose phosphate pathway [137]. The concentration of tyrosine within adult vitreous is 91 µmol/l [138]. Antioxidant activities of tyrosine, as observed in vitro include anti-lipid peroxidation, superoxide anion radical scavenging, hydrogen peroxide scavenging, and metal chelating activities [137]. 

vi. Human serum albumin (HSA): HSA is an anionic globular protein with a molecular weight of approximately 69 kDa [133,139]. HSA is sourced by filtration from blood and constitutes about 80% of the average protein concentration within the healthy vitreous body [133,140]. The molecular structure of HSA confers multiple antioxidant properties on it including an ability to bind potential ROS-generating ligands (for example, the transition metals copper and iron), scavenge hydroxyl radicals through its reduced cysteine residue (Cys34), and scavenge peroxynitrite through its thiol (–SH) group (for a review of the antioxidant properties of HSA, see [141]).

vii. Transferrin: Transferrin (molecular weight ~80 kDa) is a glycoprotein with two specific high-affinity binding sites for iron [142,143]. Vitreous contains a mean transferrin concentration of 0.0878 g/L [22]. As an antioxidant, transferrin is an iron chelator which keeps ionic iron sequestered at physiological PH and minimizes the involvement of iron in iron-dependent radical reactions [144]. This property helps to reduce intravitreal iron toxicity during vitreous haemorrhage [145].

viii. Pigment Epithelium-Derived Factor (PEDF): PEDF is a 50 kDa glycoprotein, a member of the serine protease inhibitors, which is produced by the retinal pigment epithelium [2]. Mean concentration of PEDF has been shown to be higher in vitreous from diabetic macular oedema patients (2.03 µg/mL) compared to normal vitreous (0.83 µg/mL) [146]. Also, PEDF has been detected by proteomic and western blot analyses in vitreous of normal and PDR patients, with a downward regulation of PEDF in vitreous of PDR patients [134]. A more recent study, however, has indicated that PEDF is absent in normal vitreous but present in vitreous during ischemic retinopathies [147]. PEDF exerts anti-angiogenic activity within the eye, and its loss has also been implicated in the pathogenesis of AMD [148]. 

#### 4.1.3. Trace Elements: Two Trace Elements Detected in Human Vitreous Are Selenium and Zinc

i. Selenium: Selenium is an essential trace element found within both adult and infant vitreous, with an average concentration of 0.1035 µmol/L [22]. A trend of higher concentrations of selenium has been reported in adult male vitreous compared to the female [149]. High selenium rich sources include sea foods, meat products, and cereals. Low levels sources include milk, fruits, and vegetables [124,150]. Selenium can exist in biological systems as a selenoprotein (an enzymatic antioxidant; e.g., selenoprotein P and glutathione peroxidase), an organic selenium compound (examples include selenomethionine and dimethyselenide) or inorganic forms (as selenites and selenates) [151]. Selenium functions indirectly as an antioxidant through its incorporation in antioxidant enzymes, selenoenzymes [149].

ii. Zinc: Zinc is the second most essential trace metal in the body and the most abundant within the eye [152,153]. Zinc has been detected in both adult and infant vitreous [22,154]. Zinc concentration within adult human vitreous approximates to 1.95 µmol/L [22]. While its specific roles within vitreous are yet to be elucidated, zinc is known to exert its antioxidative effects by protecting sulfhydryl groups from oxidation. Also, zinc acts as a stimulus for the synthesis of the cysteine-rich, metal-binding protein, metallothionein [153]. Metallothionein functions as a scavenger for damaging oxygen free radicals (e.g., hydroxyl radicals) and protects tissues from various forms of oxidative injury including lipid peroxidation and glycoxidation (a phenomenon which can result in vitreous degeneration) [155]. In fact, in Eales’ disease, where oxidative stress has been implicated as a potential causative mechanism, studies have reported reduced levels of zinc and increased levels of oxidation and peroxidation products within vitreous [121,153].

#### 4.1.4. Uric Acid

Uric acid, a degradation product of the metabolic breakdown of purine nucleotides, functions as an antioxidant at normal concentrations. In the presence of oxidative stress, however, there is upregulation of UA concentrations and a concurrent shift in redox balance, causing UA to become oxidant [156,157]. As a water-soluble physiological antioxidant, UA reacts highly with peroxyl or hydroxyl radicals to yield urate, its intermediate radical, which is subsequently reduced by ascorbate as part of an overall antioxidant effect [158,159]. In bovine vitreous, the concentration of UA is 170 µM [160]. Intravitreal UA levels of 156–170 µmol/L have been reported for subjects with diabetic macular oedema, 3-fold higher than non-diabetic controls (52–70 µmol/L) [156,161].

### 4.2. Enzymatic Vitreous Antioxidants

The antioxidant enzymes detected in vitreous are superoxide dismutase, glutathione peroxidase, and catalase.

a. Superoxide dismutase (SOD): SOD is a metalloprotein enzyme that catalyzes superoxide radicals to hydrogen peroxide and molecular oxygen [165]. SOD is comprised of three isoforms: cytosolic SOD (SOD1), mitochondrial SOD (SOD2), and extracellular SOD (SOD3) [166,167,168]. SOD1 and SOD3 are copper-and-zinc-containing SOD (Cu/Zn-SOD) whereas SOD2 is a manganese-containing SOD (Mn-SOD) [17,165]. SOD3 isoenzyme, an interstitially located, homotetrameric, Cu/Zn-containing SOD, is distinctively concentrated at the vitreous base and cortex [165,169]. SOD3 interacts with specific proteoglycans at the vitreous base and cortex, and functions to regulate oxidative stress response in vitreous and to prevent oxidative damage to the adjacent neural retina [169]. Thus, dysregulation of SOD3 activity at the vitreous base may be part of the pathophysiological mechanism for oxidative-stress-related vitreo-retinal pathologies such as diabetic vitreo-retinopathy [169]. Average intravitreal concentrations of SOD reported in Eales’ disease and diabetic vitreous haemorrhage are 0.9 and 22.1 IU/mg protein [121].

b. Glutathione peroxidase (GPX): Of the five isoenzymes belonging to this family of selenoenzymes, the extracellular GPX3 and phospholipid GPx4 are found within the vitreous body [170]. As a homotetrameric protein, GPX3 catalyzes the reduction of organic hydroperoxides and hydrogen peroxides (H_2_O_2_) to alcohol and water by employing GSH as an electron donor. GPX4, a monomeric protein, is capable of directly reducing phospholipid and cholesterol hydroperoxides [171]. However, evidence from bioanalytical studies of this enzyme indicates that less than 50% of GPX is active within vitreous. Also, the antioxidant enzyme activity of GPX has been attributed to the tetrameric form and not the monomeric [170]. This antioxidant activity depends on the availability of reduced GSH. 

c. Catalase: Catalase is a tetrahedral hemoprotein that protects tissues from the toxic effects of peroxide by converting peroxides into water and oxygen [172]. The human vitreous body has an average concentration of 58 µl O_2_ per mg soluble protein of catalase [164]. Catalase has been detected in vitreous of PDR patients leading to the suggestion that catalase may be a potential candidate for the treatment of acute ischemic diseases of the retina, although this association requires significant further investigation [113].

## 5. Conclusions and Future Strategies

The vitreous body is laden with antioxidant molecules that could protect against oxidative stress and diseases of vitreous as well as surrounding tissues. Concerning the former, vitreous gel liquefaction and degeneration may be due, at least in part, to depletion of vitreous antioxidants initiating gel liquefaction. Concerning the latter, deficient vitreous antioxidant capacity might contribute to chronic diseases such as cataracts, glaucoma, diabetic vitreo-retinopathy, and age-related macular degeneration. Thus, future strategies might include administration of exogenous micronutrients such as ascorbic acid, hesperidin, zinc, leucocyanidin, l-lysine, and verbascosides which have been shown to have inhibitory effects on the proposed mechanisms of vitreous degeneration, albeit only indirectly and not in vivo, certainly not in humans [173,174,175,176,177,178]. 

In terms of the role of exogenous micronutrients to augment vitreous, an important aspect to consider relates to the currently unproven efficacy of increasing intravitreal levels of exogenous micronutrients and the mode of delivery of these nutrients into vitreous. To date, there is limited data available to help explain this process, so we can only conjecture based on evidence from toxicology studies. Fluorometry studies and post-mortem toxicological analysis have shown that transfer of molecules from systemic circulation into vitreous are mediated by diffusion, hydrostatic and osmotic pressure gradients, convection, and active transport, through the blood-aqueous and blood–retina barriers [30,98,179,180,181]. Given that some of the aforesaid exogenous nutrients have been previously detected in human vitreous, one can theorize that these nutrients utilize the above-mentioned pathways to accumulate in vitreous, in spite of the fact that specific delivery channels have not been isolated for most of these nutrients [22,99,182,183]. Also, the potencies of these putative channels are unknown, and thus, achieving sufficient therapeutic doses of exogenous nutrients within vitreous may require repeated long-term administration of these agents [184]. However, to guard against systemic adverse effects due to chronic use and to ensure safety, concentrations of exogenous micronutrients administered should be the daily *Dietary Reference Intake* values for these nutrients [185]. In the case where a recommended value is not available for a micronutrient, the concentration to be consumed should be guided by data on the adverse effects observed with different concentrations of the same micronutrient.

## Figures and Tables

**Table 1:** Table showing the concentrations of antioxidant molecules in human, animal, and diseased vitreous from previous studies.

Antioxidant	Author	Human Vitreous *	Animal Vitreous	Vitreous from Diseased Eye
Ascorbic Acid	Duarte & Lunec [99] McGahan [162] Park et al. [100]	2 mmol/L 172.7 µg/mL	0.43 mmol/kg—rabbit	19.1 µg/mL—PDR
Riboflavin	Philpot & Pirie [104] Long [105]		0.8 µg/ 100 mL—ox 8.0 µg/L—bovine	
Glutathione	Sulochana et al. [121] Cicik et al. [117] Géhl et al. [122]	0.26 mmol/L 2.35 µmol/µg protein		2.8 µg/mg protein—ED 17.7 µg/mg protein—DVH 0.58 µmol/L—PDR 15.7 µmol/L—PVR 4.54 µmol/µg protein—PDR
Taurine	Diederen et al. [163] Heinämäki et al. [125]	22.6 µM	1.72 µmol/mL	26.0 µM—RRD 28.1 µM—PDR
Uric acid	Sebag [160] Krizova et al. [156,161]	156–170 µmol/L	170 µM—bovine	52–70 µmol/L—DMO
Tyrosine	Shih [138]	91 µmol/L		
Transferrin	Kokavec [22]	0.0878 g/L		
Selenium	Kokavec [22]	0.1035µmol/L		
Zinc	Kokavec [22]	1.95µmol/L		
Superoxide dismutase	Sulochana et al. [121]			0.9 IU/mg protein—ED 22.1 IU/mg protein—DVH
Glutathione peroxidase	Sulochana et al. [121]			0.61 µmol of GSH utilized/mg protein/min—ED 0.49 µmol of GSH utilized/mg protein/min—DVH
Catalase	Mayer [164]	58 µL O_2_/mg protein		
PEDF	Ouchi et al. [146]	0.83 µg/mL		2.03 µg/mL—DME

*, Samples used were from cadaver or eyes undergoing vitrectomy for idiopathic macular holes or epiretinal membranes; ED, Eales’ disease; DVH, diabetic vitreous haemorrhage; PDR, proliferative diabetic retinopathy; PVR, proliferative vitreo-retinopathy; RRD, rhegmatogenous retinal detachment; DMO, diabetic macular oedema; GSH, reduced glutathione; PEDF, pigment epithelium-derived factor.
